# The Therapeutic Effects of Whole-Body Vibration in Patients With Fibromyalgia. A Randomized Controlled Trial

**DOI:** 10.3389/fneur.2021.658383

**Published:** 2021-06-02

**Authors:** José A. Mingorance, Pedro Montoya, José G. Vivas Miranda, Inmaculada Riquelme

**Affiliations:** ^1^Department of Nursing and Physiotherapy, University of Balearic Islands, Palma, Spain; ^2^University Institute of Health Sciences Research (IUNICS), University of Balearic Islands, Palma, Spain; ^3^Laboratory of Biosystems, Institute of Physics, Federal University of Bahia, Salvador, Brazil

**Keywords:** fibromyalgia, balance, whole-body vibration, pain sensitivity, motor performance

## Abstract

Fibromyalgia is a chronic pain disease with few effective therapeutic options. We evaluated the efficacy of a 12-weeks therapy program that involves the use of whole body vibration in patients with fibromyalgia. The experimental group (*N* = 20 patients) participated in a neuromuscular training with a rotational whole body vibration platform for 12 weeks. The control group (*N* = 20 patients) received no physiotherapy treatment. The following variables were assessed before, after and 3 months after the therapy program: Fibromyalgia impact questionnaire, pain intensity, quality of life, sensitivity measurements (pressure pain thresholds, vibration thresholds), motor function tasks (Berg scale, 6-min walk test, isometric back muscle strength), and static and dynamic balance. We found improvements in the indexes of functional disability, static equilibrium and vibration sensitivity and a reduction of pain sensitivity. Our results showed that the intervention group improved almost all parameters immediately after the therapy program, in contrast to the control group that showed no changes. These improvements were not maintained in the follow-up. The whole body vibration therapy can be an effective therapy in patients with fibromyalgia and it can improve symptomatology and quality of life in these patients.

## Introduction

Fibromyalgia (FM) is a chronic syndrome characterized by widespread pain sensitivity, fatigue and cognitive symptoms. It affects 3–5% of general population, women predominantly (1–3). The prevalence of fibromyalgia in Spain is 2.4%. The Balearic Islands have about 20,000 people affected by fibromyalgia, with a 21:1 female/male ratio (4). It has few effective therapeutic options available. For this reason, it is necessary to adopt effective treatments able to reduce its symptomatology. Neuromodulator agents, antidepressants, or muscle relaxants are the most successful pharmacotherapies in its treatment (5, 6). Recent reviews have exposed rehabilitation techniques, such as massage, myofascial release or acupuncture or multidisciplinary rehabilitation as yielding benefits at short and long term in function and multidisciplinary rehabilitation at intermediate term in pain (7, 8). Clinical practice guidelines consider physical exercise and cognitive-behavioral therapy as the best non-pharmacological interventions (9). Exercise yields functional and pain improvement at short and intermediate term (8). Global aerobic and strength exercises seem similar and better than stretching exercises alone (10). Some studies have proposed different therapies like individualized moderate-intensity exercise (11, 12) or Tai-chi (13) with good results (14).

The whole body vibration (WBV) therapy has proven to improve health status (15–18), strength (19, 20), static and dynamic balance (21), physical function (20), pain and quality of life (22, 23). To the best of our knowledge, all the previous studies combined WBV with an associated exercise program. Thus, it remains uncertain the active component of this therapy (24). Some reviews highlighted the need for further research in this field to improve understanding of the effects of WBV in patients with fibromyalgia (9, 25). Furthermore, these previous studies did not measure important parameters such as motor function, proprioception or vibration sensitive threshold. The objective of this randomized controlled trial was to compare the effects of a 12-weeks therapy program consisting in the use of WBV with a control group, which did not perform any therapy program. Our main hypothesis is that body vibration therapy is a useful treatment in fibromyalgia patients to improve pain. In addition, we hypotetize that WBV is effective in improving a wider range of symptoms, such as health-related quality of life, fibromialgia impact, sensitivity and motor parameters, such as muscle strength, balance and motor performance.

## Materials and Methods

### Patients

Forty patients with fibromyalgia, diagnosed by rheumatologists according to the American College of Rheumatology 2016 criteria (3), were recruited from the Fibromyalgia Patients Association in Palma de Mallorca (Spain) by an external researcher. All patients met the inclusion criteria: (A) age between 30 and 65 years, and (B) diagnosis of fibromyalgia according with the American College of Rheumatology (3) and with negative tests for all overlapping fibromyalgia symptoms (multiple sclerosis, hypothyroidism, anemia, autoimmune diseases of the connective tissue, small-fiber polyneuropathy, etc.). Exclusion criteria were history of severe trauma, peripheral nerve entrapment, inflammatory rheumatic diseases, presence of neurological or oncological disorders, osteoporosis, pregnancy, serious cardiovascular disease, pacemaker, hip and knee implants, participation in a psychological or physical therapy program, participation in regular physical exercise more than once a week over a 2-weeks period in the last 5 years.

### Procedure

A single blind randomized controlled trial was performed. The Ethics Committee of the Balearic Islands approved the study (IB-2586/15 PI) and it was registered at ClinicalTrials.gov (NCT03782181). Randomization was performed in two stages: generation of numbers and blind allocation. The envelopes indicated which group the participants would be included in were opened after informed consent was obtained. It was not possible to blind therapists, but the outcome assessors were not informed about the allocation of patients in the respective groups.

We used a 1:1 allocation ratio and the 40 participants were randomly allocated into one of the groups. A single-blinded, 1:1 parallel-group, randomized controlled trial was conducted. On the day of admission, a physiotherapist informed patients about the study and asked them to participate. A randomization list was created by a statistician and administered by a secretary in sequentially numbered, sealed envelopes. There was a commitment to adherence to be able to complete the study along with a flexible schedule so that all patients could come to all sessions. Patients did not report any side effects during treatment.

The intervention group performed a program designed with the parameters previously recommended by Chulvi-Medrano et al. (23). The program consisted of maintaining three different positions on the platform during vibration: standing with both feet on the platform for 45 s, unilateral static position, 22 s with each leg. These sessions were held individually in the gymnasium of the Fibromyalgia Association in Palma and the entire program was supervised by a physiotherapist trained in WBV. Participants in the control group did not perform any program. The intervention took place during the spring of 2019, and 3 months after treatment (follow-up), patients completed the same initial questionnaires.

Two types of platforms can deliver WBV. One is a vertical vibration device that induces oscillations over a vertical axis. The second one is a rotational vibration device that induces reciprocal displacements on the left and right sides of a fulcrum. Some studies using vertical vibration have shown an improvement in the anteroposterior stability (19), while studies using rotational vibration showed an improvement of the mediolateral stability, more related to the risk of falls (20). We opted for using the rotational vibration and WBV was programmed according with the parameters published by previous studies (23): 25 Hz of frequency, and 3 mm of amplitude. None of the patients performed physical activity or received physiotherapy treatment before or during the intervention and follow-up periods.

### Assessment

Assessment of all outcomes in both groups was undertaken at baseline, immediately after the therapy and at follow-up (3 months later).

#### Self-Report Questionnaires

- *Fibromyalgia impact questionnaire*. This is a validated instrument designed to quantify the overall impact of fibromyalgia. We used the Spanish version of the questionnaire (26). Higher scores mean poor functional status.
- *Visual analog pain scale* (27). Each participant was asked to indicate their current level of pain using a 20 cm visual analog scale ranging from 0 to 100 (unbearable pain). This has been reported to be a reliable method for assessing pain (28).
- *Quality of Life Index* (27). This is a self-report questionnaire, previously used to assess quality of life in patients with fibromyalgia (29). We used the Spanish version (27). A higher score is indicative of a higher quality of life.

#### Sensitivity measures

- *Pressure pain sensitivity* was assessed by means of the measure of pressure pain thresholds, expressed in Newtons. This method has demonstrated its reliability to assess pain sensitivity (30, 31). Pressure stimuli were applied on two bilateral body locations: epicondyles and index finger. The pressure pain threshold was defined as the pressure value considered as painful by the participant.
- *Vibration thresholds* were evaluated by using a Vibratron (Physitemp Instruments), which consists of a controller and two transducers used to determine the intensity of the vibratory stimulus perceived by the patient. The testing started with an intensity above the threshold, and then it was gradually reduced, asking participants to indicate when the vibration was not perceived. Vibration values displayed on the control unit are the amplitude of vibration, proportional to the square of applied voltage (32, 33).

#### Motor Function Tasks

- *Berg scale*. This is a functional assessment tool, consisting of 14 functional tasks. The general scores range from 0 to 56 (highest level of function). It has been previously used in patients with fibromyalgia to assess balance (34). Patients must complete 14 tasks while the examiner rates the patient's performance on each task. Test items are representative of daily activities that require balance, such as sitting, standing, bending over, and stepping. It does not include the assessment of gait.
- *Six-minute walking test*. This is a functional test in which the patient walks as far as possible for 6 min (35). It has been validated for patients with fibromyalgia (34, 36).
- *Isometric back muscle strength was* determined by a dynamometer (T.K.K.5002). Participants were asked to pull extending their back, trying to put the body as vertical as possible. This test has proven to be reliable in the assessment of back muscle strength (22).

#### Static and Dynamic Balance

- *Static balance* was assessed by using a modified version of the Romberg's test (37). Postural control is dependent on input from three peripheral modalities: vision, vestibular apparatus, and proprioception. Asking the participants to close their eyes during the Romberg's test helps to uncover any disordered proprioception. We analyzed the body sway during the test performance. Participants were situated below a webcam (© Logitech) placed at a mean distance of 50 cm from the participant's head. The participant was asked to remain in orthostatic position with feet parallels, arms extended along the body and eyes closed for 1 min (38). Velocity and body sway in the anterior-posterior and medial-lateral directions were extracted and analyzed by the software CvMob, which produces similar results than posturography (39).
- *Dynamic balance* was assessed by means of a gait task. Participants were instructed to walk on a 4 m carpet at their normal walking step. Optical markers were attached at the lateral condyle of the femur, great trochanter and lateral malleolus. Subject's motion was digitally recorded with a video camera at 210 frames per second (CasioExilimEX-FS10). The camera was positioned at 4 m from the carpet. Changes in position and velocity along the x-axis were obtained by CvMob (37, 39), analyzing the following measures: mean sway velocity, mediolateral and anteroposterior body sway, gait speed, stride length, and percentages of time in the stance and swing phases.

### Statistical Analyses

The calculation of the sample size was carried out using the G^*^Power software (40) and taking Visual Analog Scale of pain as primary outcome. Pain was measured with VAS and we looked for change in VAS score from baseline. The assumption we did was that the VAS score in WBV group was reduced more compared to the control group. Two paired averages (repeated in two groups) and bilateral contrast was used, with an alpha risk of 0.05 (Z1-α = 0.05). The difference to be detected between groups in the average of the changes of Visual Analog Scale of Pain was −10 mm. A standard deviation of 9 was used. Our assumption was: Change in pain in WBV group −10 millimeters (SD 9) and control group −0 millimeters (SD 9). The effect size d was 1.11. Accepting an alpha risk of 0.05 in a bilateral contrast, 19 subjects in the intervention group and 19 in the control group were needed to detect a difference equal to or >−10 mm in VAS pain score.

As Kolmogorov-Smirnov tests confirmed the normal distribution of the variables across the categories (all *p* > 0.172), two-way analysis of variance were performed, with the between-factor GROUP and the within-subject factor TIME. Effect size was calculated by means of the Cohen's d and it was interpreted as small (~0.25), medium (~0.5), or large (>0.8). The significance level was set at 0.05. All the analyses were performed using SPSS Statistics.

## Results

The 40 subjects enrolled in the groups adhered totally to the program, with no occurrence of sample loss. The mean age was 52.5 ± 8.3 years. Most of the participants were female (90%). The mean duration of pain was 7.3 years, with an average of 3 years for the clinical diagnosis of fibromyalgia.

Table 1 displays patients characteristics of both groups. Groups did not differ in their sociodemographic characteristics or in the baseline scores of the assessment variables (all *p* > 0.05). For medical reasons, medication was not discontinued during the study. The most frequent medication taken by the participants was: Duloxetine (90% of the patients), taken as a capsule in the morning with a dose of 60 mg; Milnacipran (85% of the patients), used in doses of 100 mg per day; Pregabalin (80%), taken as capsules twice a day with a dose of 300 mg; and Cyclobenzaprine, taken as capsules once a day with a dose of 30 mg (20%).

**Table 1:** Sociodemographic data from the control and intervention group.

	**Control group (*N =* 20)**	**Intervention group (*N =* 20)**	
	**Mean ± sd**	**Mean ± sd**	***p***
Age (years)	50.25 ± 8.53	52.30 ± 8.04	0.43
BMI (Kg/m^2^)	23.34 ± 1.23	22.95 ± 1.30	0.34
Height (centimeters)	169.15 ± 6.41	168.25 ± 6.35	0.65
Weight (Kilograms)	67.00 ± 7.46	65.05 ± 5.82	0.36
Pain time (years)	7.50 ± 3.22	6.75 ± 2.29	0.40
Gender (women)	*N =* 19	*N =* 19	0.15

*Sd, Standard deviation; BMI, body mass index*.

### Self-Report Questionnaires

**Figure 1:**
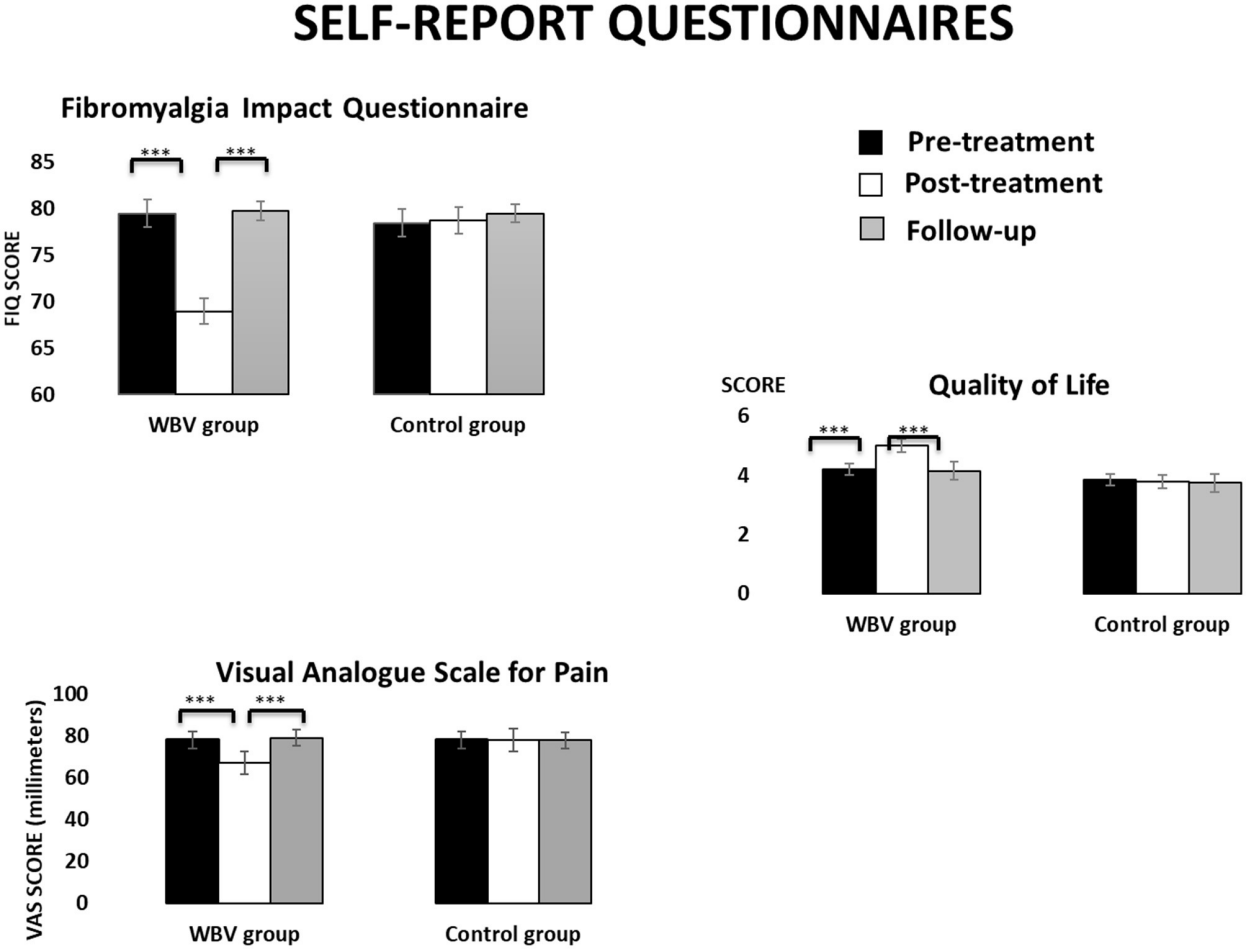
Descriptive data (mean and TE) for the WBV group and the control group in self-report questionnaires at the three assessment times.

Figure 1 displays the descriptive data in the three assessment times for each group in self-report questionnaires.

- *Fibromyalgia impact questionnaire* scores showed a main effect TIME [*F*_(2, 76)_ =87.47, *p* < 0.001], showing lower scores in the post-treatment than in the pre-treatment or follow-up (both *p* < 0.001). An interaction effect GROUP × TIME [*F*_(2, 76)_ =88.24, *p* < 0.001], indicated that these effects were produced only in the intervention group. *Post-hoc* mean comparisons revealed that participants in the intervention group significantly decreased their scores from the pre- to the post-treatment (*p* < 0.001, Cohen's d = 5.22 and effect size of 0.93) but these scores increased from the post-treatment to the follow-up (*p* < 0.001, Cohen's d = −5.14 and effect size of −0.93).
- *Visual analog pain scale* showed a significant main effect TIME [*F*_(2, 76)_ = 40.69, *p* < 0.001] with lower scores in the post-treatment than in the pre-treatment or follow-up (both *p* < 0.001). An interaction effect GROUP × TIME [*F*_(2, 76)_ = 41.34, *p* < 0.001] indicated that only the intervention group reported lower pain scores in the post-treatment and follow-up compared to the pre-treatment (both *p* < 0.01, Cohen's d >8.30 and effect size >0.97), although scores in the follow-up were higher than in the post-treatment (*p* < 0.001, Cohen's d = −5.94 and effect size of −0.95). Moreover, the intervention group showed higher scores than the control group in the pre-treatment (*p* = 0.006) but lower scores in the post-treatment (*p* = 0.005). No differences between groups were found in the follow-up.
- In the *Quality of Life Index* a significant interaction effect GROUP × TIME [*F*_(2, 76)_ = 19.65, *p* < 0.001] revealed that the intervention group perceived higher quality of life in the post-treatment than in the pre-treatment (*p* < 0.001, Cohen's d = −3.55, and effect size of −0.87), although it decreased again between the post-treatment and the follow-up (*p* < 0.001, Cohen's d = 3.67 and effect size of 0.87). No significant changes were observed in the control group. Main effects TIME [*F*_(2, 76)_ = 20.08, *p* < 0.001] and GROUP [*F*_(2, 76)_ = 20.08, *p* < 0.001] showed the same time pattern and indicated higher quality of life in the intervention group than in the control (*p* = 0.045).

### Sensitivity Measures

**Figure 2:**
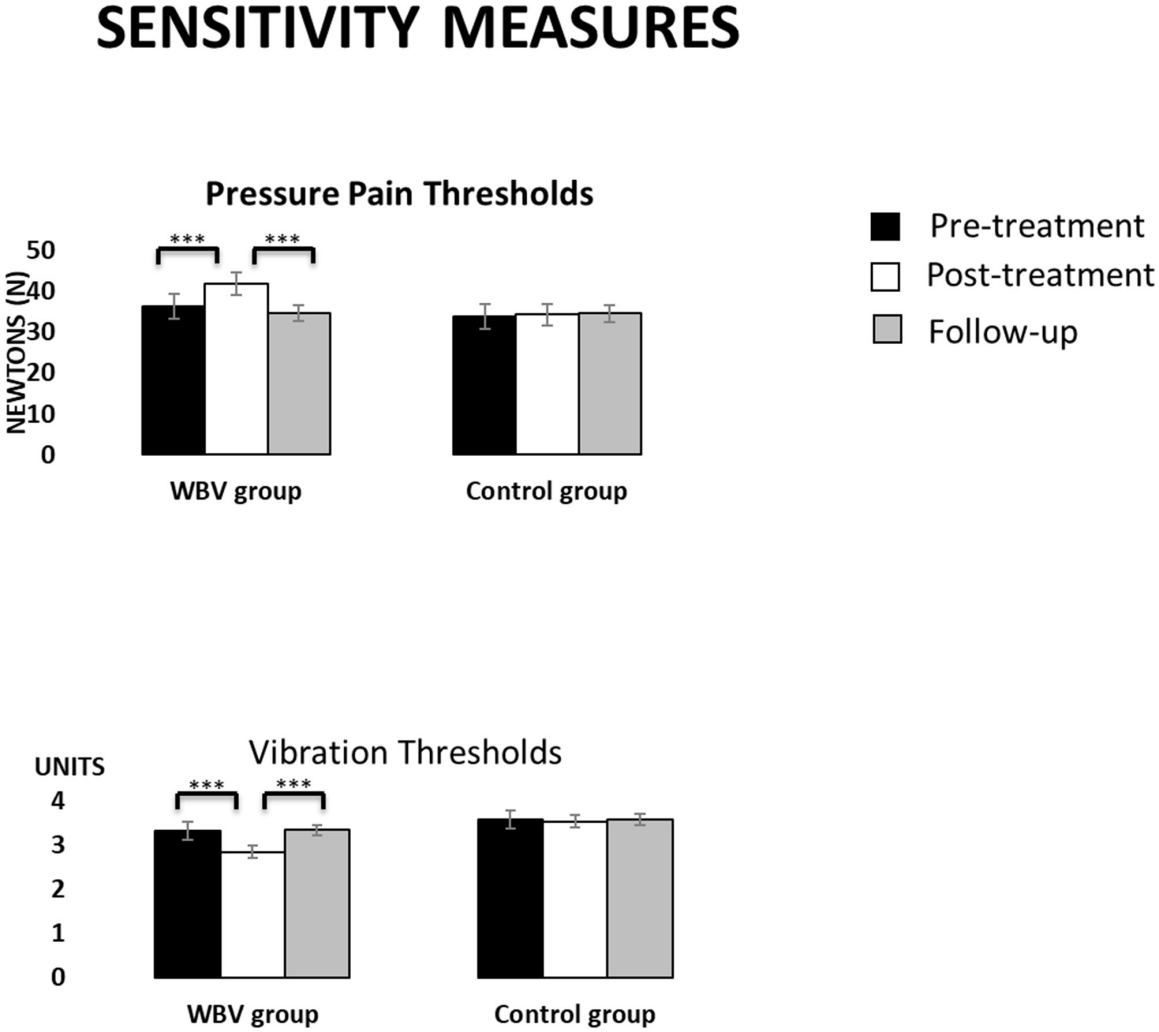
Descriptive data (mean and TE) for the WBV group and the control group in sensitivity measures at the three assessment times. ***p < 0.001.

Figure 2 displays the descriptive data of sensitivity measures in the three assessment times for each group.

- For *pressure pain thresholds*, interaction effects GROUP × TIME in both body locations (both F > 38.50, both *p* < 0.001) indicated an increasing of thresholds from the pre-treatment to the post-treatment (all *p* < 0.001, all Cohen's d > −2.45 and effect size >−0.77) only in the intervention group. Nevertheless, thresholds decreased again from the post-treatment to the follow-up (all *p* < 0.001, Cohen's d = 2.33 and effect size of 0.76).
- *Vibration thresholds* showed interaction effects GROUP × TIME in both body locations (both F > 27.60, both *p* < 0.001) indicating a reduction of thresholds from the pre-treatment to the post-treatment (all *p* < 0.001, all Cohen's d > 2.92 and effect size >0.82) and an increment from the post-treatment to the follow-up (all *p* < 0.001, Cohen's d > −2.91 and effect size >−0.82) only in the intervention group. In both body locations, thresholds at post-treatment were lower in the intervention than in the control (both *p* < 0.004).

### Motor Function Tasks

**Figure 3:**
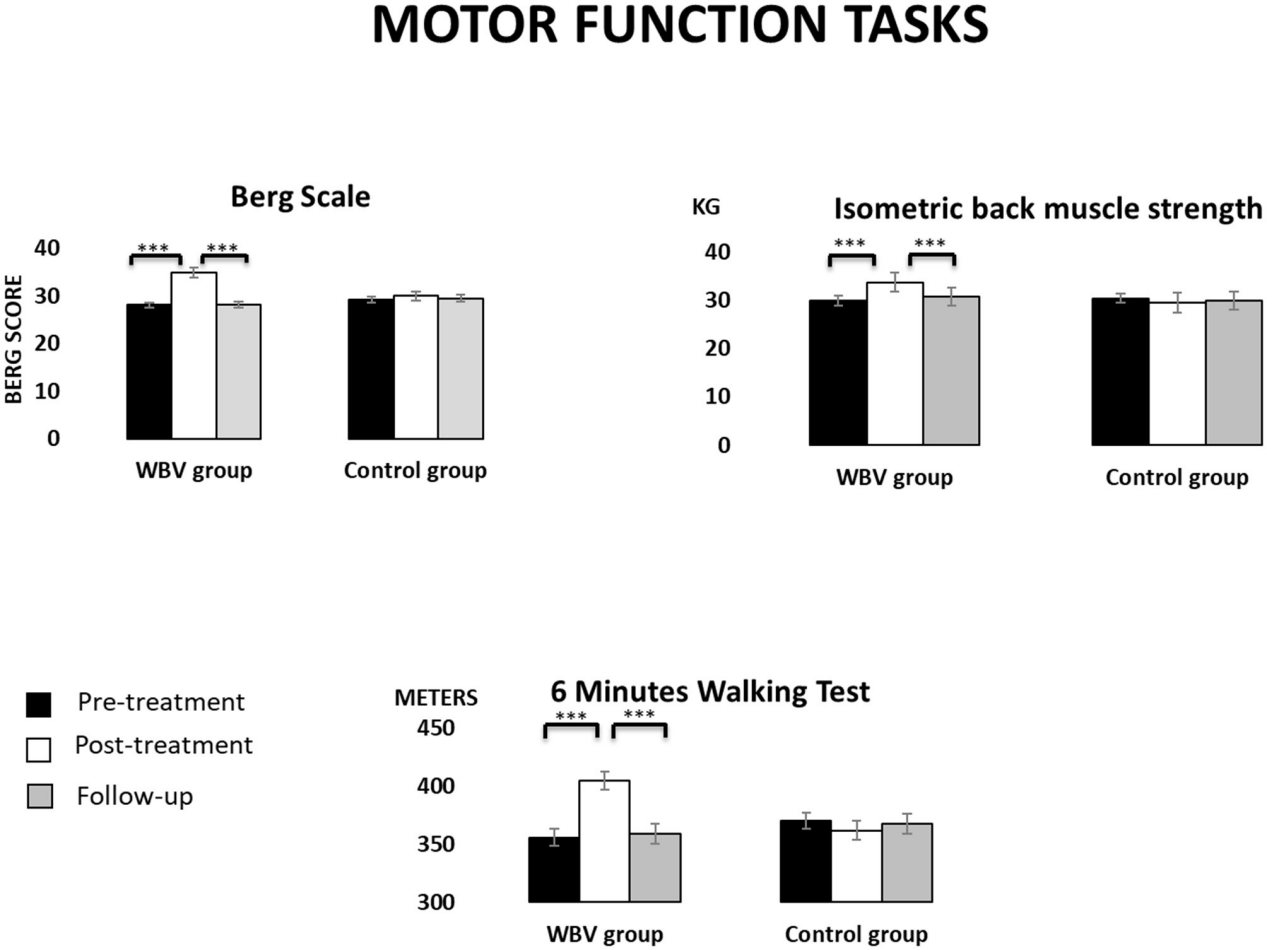
Descriptive data (mean and TE) for the WBV group and the control group in motor function tasks at the three assessment times. ***p < 0.001.

Figure 3 displays the descriptive data of motor function tasks in the three assessment times for each group.

- The *Berg scale* showed a significant interaction effect GROUP × TIME [*F*_(2, 76)_ = 73.60, *p* < 0.001]. *Post-hoc* mean comparisons revealed that participants in the intervention group significantly improved their scores from the pre- to the post-treatment (*p* < 0.001, Cohen's d = −7.19 and effect size of −0.96) but these scores decreased from the post-treatment to the follow-up (*p* < 0.001, Cohen's d =7.17, and effect size of 0.96). Moreover, the intervention group showed higher scores than the control group in the post-treatment (*p* = 0.001). Main effects TIME [*F*_(2, 76)_ =106.32, *p* < 0.001] confirmed this time pattern.
- The *6-min walking test* showed significant effects TIME [*F*_(2, 76)_ =16.56, *p* < 0.001] and GROUP × TIME [*F*_(2, 76)_ = 34.87, *p* < 0.001]. *Post-hoc* mean comparisons revealed that participants in the intervention group significantly improved their scores from the pre- to the post-treatment (*p* < 0.001, Cohen's d = −4.00 and effect size of −0.89), although these scores decreased from the post-treatment to the follow-up (*p* < 0.001, Cohen's d = 3.76 and effect size of 0.88). No significant changes were observed in the control group.
- *Isometric back muscle strength* also showed significant effects TIME [*F*_(2, 76)_ = 5.95, *p* = 0.009] and GROUP × TIME [*F*_(2, 76)_ = 13.90, *p* < 0.001]. *Post-hoc* mean comparisons revealed that participants in the intervention group significantly improved their back muscle strength from the pre- to the post-treatment (*p* < 0.001, Cohen's d = −2.17 and effect size of −0.73) but this strength decreased from the post-treatment to the follow-up (*p* = 0.001, Cohen's d = 1.62 and effect size of 0.63).

#### Static and Dynamic Balance

**Figure 4:**
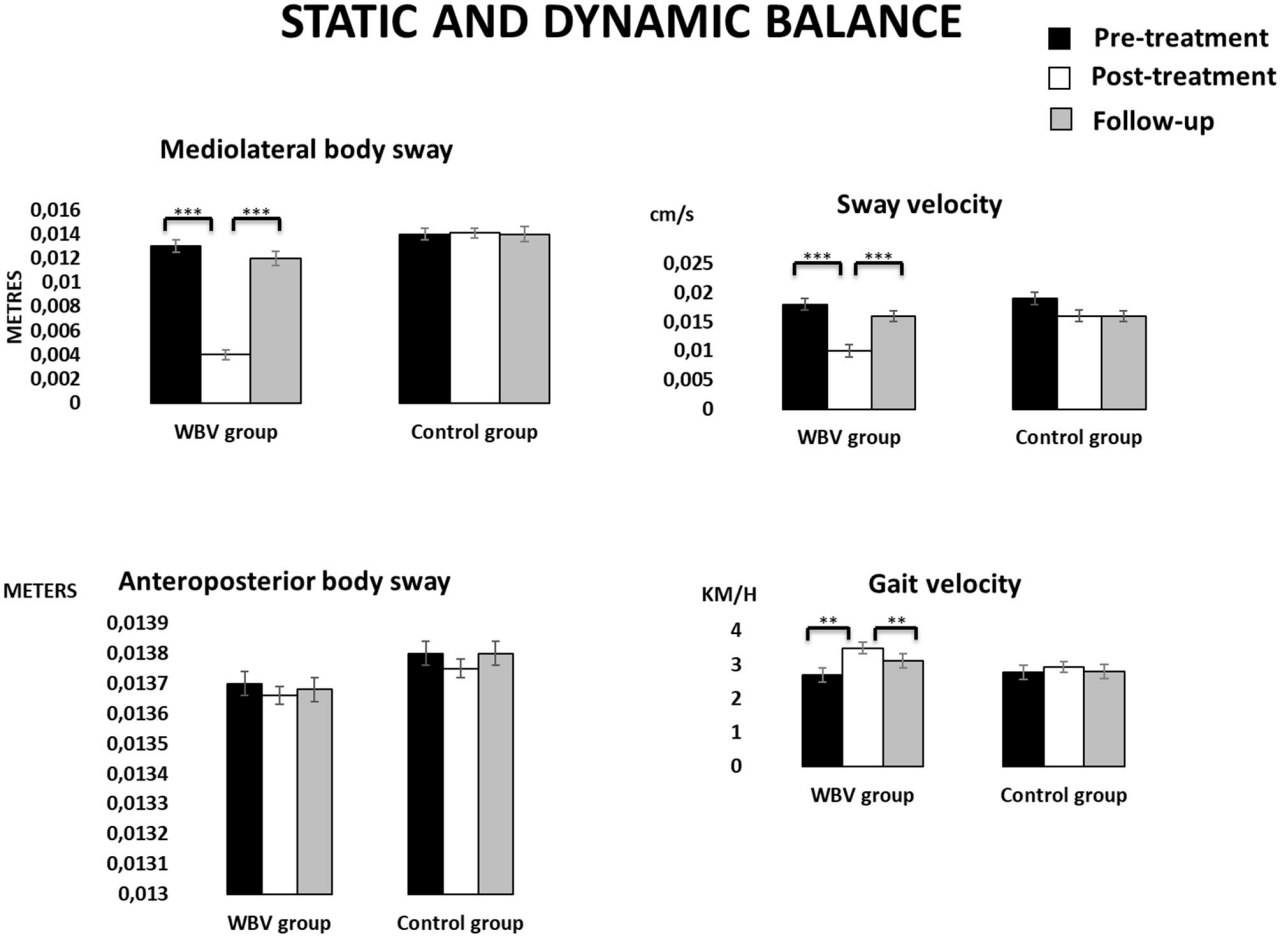
Descriptive data (mean and TE) for the WBV group and the control group in static and dynamic balance at the three assessment times. ***p < 0.001, **p < 0.001.

**Figure 5:**
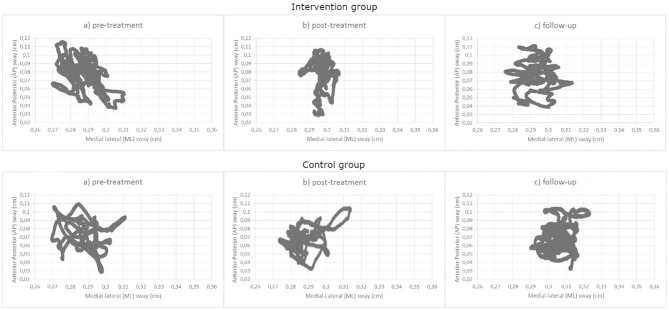
Mean of medial-lateral body sway (axis X) and anteroposterior body sway (axis Y) for the WBV group and the control group at the three assessment times.

Figures 4, 5 displays the descriptive data of balance in the three assessment times for each group.

- In *static balance*, sway in the medial-lateral axis, as well as mean sway velocity showed significant effects TIME (all F > 5.83, all *p* < 0.01) and GROUP × TIME (all F > 3.34, all *p* < 0.05). Again, *Post-hoc* mean comparisons revealed that participants in the intervention group increased significantly their mean velocity values and decreased their sway values from the pre- to the post-treatment (all *p* < 0.001, all Cohen's d >5, all effect size >0.78), although these improvements were reduced between the post-treatment and the follow-up (all *p* > 0.05, Cohen's d > −3.5, effect size >−0.87). The intervention group displayed slower and shorter sways than the control group only in the post-treatment assessment (all *p* < 0.01). No significant statistical effects were found in the anteroposterior sway.
- Regarding the dynamic balance, no significant differences were found in any of the parameters except gait speed, where a main effect TIME [*F*_(2, 76)_ = 13.88, *p* < 0.001] and an interaction effect GROUP × TIME [*F*_(2, 76)_ = 3.34, *p* = 0.047] revealed again an improvement only in the intervention group between the pre- and the post-treatment, and a decline between the post-treatment and the follow-up (both *p* < 0.001, Cohen's d = −3.28 and 1.37, effect size of −0.85 and 0.57, respectively). The intervention group was faster than the control group in the post-treatment (*p* = 0.008)

## Discussion

The present study analyzed the effects of a 12-weeks WBV program, in comparison with a control group that did not perform any training. The program showed improvements after the treatment in all parameters, although the benefits were not maintained in the follow-up. According to our knowledge, it is the first study to assess only the effect of WBV without combination with an exercise program and adds a follow-up to assess whether the effect achieved is maintained or not over time.

Our findings are like those reported previously indicating that WBV combined with an exercise program enhanced balance and muscle strength (19–21), reduced pain and increased quality of life in patients with fibromyalgia (16, 17, 20, 22, 23)]. Our study further revealed changes in somatosensory perception, improvements in motor function and a reduction of clinical comorbidities (12, 34).

Improvements in the indexes of functional disability, balance and vibration sensitivity were produced along with the decrease of pain. This is of paramount importance, as traditional exercise protocols based on moderate-intensity exercise, did not impact pain in patients with fibromyalgia (41, 42). The primary sensory inputs used for orientation in space are those provided by proprioceptive receptors such as neuromuscular spindles, the Golgi apparatus and skin afferents. In chronic pain, an abnormal perception of pain has been observed in light somatosensory stimulation, indicating a relationship between pain and spatial orientation (15). It has been suggested that pain can affect the mechanisms of postural control (12), leading to deterioration in the body's anticipatory postural adjustments (43). The relationship between pain sensitivity and sensitivity to other somatosensory inputs has been established (44). Further research should establish if a somatosensory therapy, such as WBV, can be effective in eliciting changes in central somatosensory processing and be useful for future neuromodulator treatment of chronic pain.

Even though there is no consensus on the mechanism by which vibration reduce pain, some hypotheses have tried to explain its way of action. For example, the activation of A fibers produced during vibration may compete with the central and peripheral nociceptive activity in the dorsal horn of the spinal cord, resulting in a reduction of second-order nociceptive activity with the consequent decrease in the perception of pain (45–47). The observed pain reduction in the present study could be also a consequence of a heating effect, since it has been shown that friction between vibrating tissues can raise muscle temperature (48) as well as an increase in blood flow (49).

While the one-time intervention demonstrated efficacy, there was no reported long-term effect, that is, the WBV effect did not remain at follow-up. To our knowledge, this has been the only study to date to add a 3-months follow-up. This fact has shown that the physiological effects are only maintained while WBV is performed. Although we used the frequency recommended by Chulvi-Medrano et al. (23), different frequencies are commonly used to act on pain, and an increase in frequency has been observed to cause a progressive increase in EMG activity during WBV in healthy people (50). Thus, the use of different frequencies must be explored for investigating how they may affect to the maintenance of the effects.

### Strengths and Weaknesses of the Investigation

This is the first study comparing the effects of WBV without combination of an exercise program, and assessing the potential effects at a 3-months follow-up. The study has a representative sample, which includes the epidemiological percentage of men who suffer from this disease. The study has also measured parameters that other studies have not taken into account, such as functionality, static and dynamic balance, and vibration sensitive threshold.

Likewise, the study has weak points that must be considered for the correct interpretation of the results. Although the medication was controlled, it was not suppressed in the fibromyalgia participants, and opioids, tricyclics, or benzodiazepines have been shown to have side effects on the outcomes we measured. There was a lack of control of variables that could influence the results (e.g., daily physical activity). Also, more research is needed to find out how these improvements can be maintained over the time; meanwhile, WBV should be considered as a continuous or intermittent therapeutic modality (48).

## Conclusions

In conclusion, the rotational WBV seems to improve pain, functionality, quality of life and balance in people with fibromyalgia. The findings presented in this study validate the benefits of this technique, and advise clinical practitioners including this kind of rotational WBV to their treatment protocols. Further research is needed to establish the parameters assuring the maintenance of the effects and to elucidate the differences among the vibration devices, what would allow personalizing the program according to the clinical characteristics of each patient, in the spirit of personalized medicine.

## Data Availability Statement

The raw data supporting the conclusions of this article will be made available by the authors, without undue reservation.

## Ethics Statement

The study was approved by the Ethics Committee of the Balearic Islands (IB-2586/15 PI).

## Author Contributions

IR, PM, and JMin conceived and design the study. JMin performed the recruitment and data collection and supervised the intervention. JMin and JMir performed the data and statistical analyses. All authors participated in the discussion of results, writing and correction of the manuscript, and have approved the present version.

## Conflict of Interest

The authors declare that the research was conducted in the absence of any commercial or financial relationships that could be construed as a potential conflict of interest.
